# SIRT2 Deficiency Aggravates Diet-Induced Nonalcoholic Fatty Liver Disease through Modulating Gut Microbiota and Metabolites

**DOI:** 10.3390/ijms24108970

**Published:** 2023-05-18

**Authors:** Xingyu Li, Yimeng Du, Chunyuan Xue, Xiaofeng Kang, Chao Sun, Huanyan Peng, Liaoxin Fang, Yuchen Han, Xiaojie Xu, Caiyan Zhao

**Affiliations:** 1 Department of Infectious Diseases, The Third Hospital of Hebei Medical University, Shijiazhuang 050011, China; lxy0606@stu.hebmu.edu.cn; 2 Department of Genetic Engineering, Beijing Institute of Biotechnology, Beijing 100850, China; duyimeng1987@163.com (Y.D.);

**Keywords:** non-alcoholic fatty liver disease (NAFLD), non-alcoholic steatohepatitis (NASH), SIRT2, metabolomics, gut microbiota

## Abstract

Non-alcoholic fatty liver disease (NAFLD), characterized by excessive lipid accumulation in hepatocytes, is an increasing global healthcare burden. Sirtuin 2 (SIRT2) functions as a preventive molecule for NAFLD with incompletely clarified regulatory mechanisms. Metabolic changes and gut microbiota imbalance are critical to the pathogenesis of NAFLD. However, their association with SIRT2 in NAFLD progression is still unknown. Here, we report that SIRT2 knockout (KO) mice are susceptible to HFCS (high-fat/high-cholesterol/high-sucrose)-induced obesity and hepatic steatosis accompanied with an aggravated metabolic profile, which indicates SIRT2 deficiency promotes NAFLD-NASH (nonalcoholic steatohepatitis) progression. Under palmitic acid (PA), cholesterol (CHO), and high glucose (Glu) conditions, SIRT2 deficiency promotes lipid deposition and inflammation in cultured cells. Mechanically, SIRT2 deficiency induces serum metabolites alteration including upregulation of L-proline and downregulation of phosphatidylcholines (PC), lysophosphatidylcholine (LPC), and epinephrine. Furthermore, SIRT2 deficiency promotes gut microbiota dysbiosis. The microbiota composition clustered distinctly in SIRT2 KO mice with decreased *Bacteroides* and *Eubacterium*, and increased *Acetatifactor*. In clinical patients, SIRT2 is downregulated in the NALFD patients compared with healthy controls, and is associated with exacerbated progression of normal liver status to NAFLD to NASH in clinical patients. In conclusion, SIRT2 deficiency accelerates HFCS-induced NAFLD-NASH progression by inducing alteration of gut microbiota and changes of metabolites.

## 1. Introduction

Currently, non-alcoholic fatty liver disease (NAFLD) is a global healthcare burden and a major cause of morbidity [1]. A large-scale population cohort study has shown that the prevalence of NAFLD-related hepatocellular carcinoma (HCC) has increased by 4-fold in the last decade, making it one of the most rapidly growing public health problems [2]. As a clinical syndrome, NAFLD refers to excessive fat accumulation in the liver, which can range from simple steatosis, an early stage of NAFLD, to a progressed stage of non-alcoholic steatohepatitis (NASH) that manifests as hepatic inflammation and collagen deposition [3]. Eventually, NASH can progress to cirrhosis, liver failure, and even HCC. NAFLD usually occurs with concomitant other metabolic abnormities such as obesity, dyslipidemia, and insulin resistance [4]. Although multiple studies engage with the pathogenesis of NAFLD, the critical factors and specific regulatory mechanisms that affect the progress of hepatic steatosis to NASH are still not fully clarified.

In recent decades, a “multiple-hit” hypothesis was proposed to explain the pathogenesis of NAFLD, which includes various pathogenesis factors such as gut microbiota, metabolites, immunity, and genetic mutations [5]. Among them, gut microbiota is one of the primary factors contributing to the development of NAFLD [6]. Gut microbiota is symbiotic with the host and metabolizes dietary constituents in the distal colon to help the host get nutrients and energy [7,8]. The profile of gut microbiota is susceptible to dietary patterns [9], and the alteration of gut microbiota profile by high-fat/high-cholesterol (HFC) and high-fat/high-sucrose (HFS) diet has been reported [4]. In addition, diets with high fat, high cholesterol, and high sugar can increase the risk of NAFLD in human [10,11] However, the effect of high-fat/high-cholesterol/high-sucrose (HFCS) diet on gut microbiota dysbiosis remain unclear.

Sirtuin2 (SIRT2) is a member of the highly conserved nicotinamide adenine dinucleotide (NAD^+^)-dependent histone deacetylases family sirtuins (SIRTs) [12], which is highly expressed in tissues with high metabolic activity, such as heart, adipose tissue, brain, and liver [13]. Accumulating studies have reported that SIRT2 is critical for maintaining glucose and lipid metabolism homeostasis [14,15]. Additionally, SIRT2 performs inflammation regulation function, and effectively ameliorates oxidative stress and mitochondrial dysfunction [16]. An animal experiment found that SIRT2 improves NAFLD progression fed with high-fat diet (HFD) via the SIRT2-hepatocyte nuclear factor 4α (HNF4α) pathway [17]. However, the regulatory mechanism and phenotype of HFCS-induced NALFD and the interplay of SIRT2 with gut microbiota in NALFD remains unknown. 

The purpose of this study was to investigate the role that SIRT2 played in the HFCS-induced NAFLD-NASH progression through modulating gut microbiota and its metabolites. We used a HFCS diet to establish a NAFLD model in SIRT2 KO mice and their wildtype (WT) littermates, and then explored the regulatory mechanisms of SIRT2 on NAFLD through serum metabolomics and fecal 16S rRNA sequencing.

## 2. Results

### 2.1. SIRT2 Knockout Mice Are Susceptible to HFCS-Induced Obesity and Hepatic Steatosis

**Figure 1:**
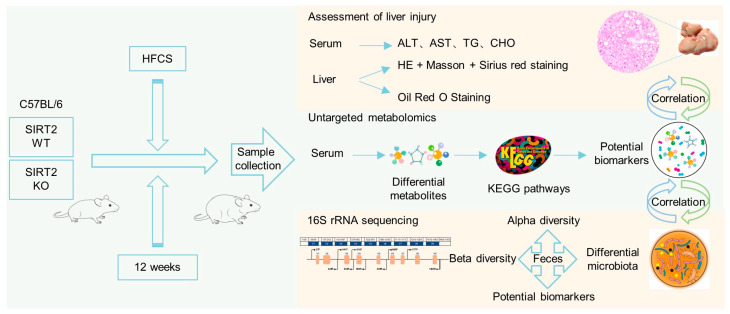
Schematic illustration of this study to investigate the effect of SIRT2 on HFCS-induced progression of NAFLD through assessment of liver injury, metabolomics analysis, and gut microbiota analysis.

**Figure 2:**
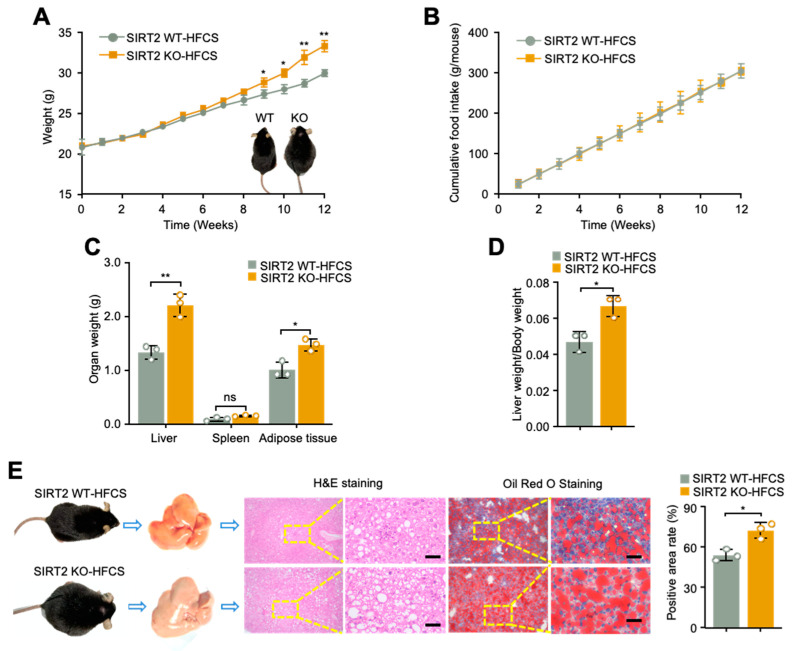
SIRT2 KO mice are susceptible to HFCS-induced obesity and hepatic steatosis. (**A**) Body weight and (**B**) food intake of SIRT2 WT and KO mice on HFCS diet (N = 9/per group). The inset of (**A**) showed representative gross morphology of the mice under the HFCS diet for 12 weeks. (**C**) Organ weight and (**D**) liver/body weight ratio of SIRT2 WT and KO mice on HFCS for 12 weeks (N = 9/per group). (**E**) Representative gross morphology, microscopic features, and H&E staining and Oil Red O staining images of liver tissues from SIRT2 WT and KO mice on HFCS for 12 weeks (scale bar, 100 μm). Quantitative analysis of Oil Red O staining was illustrated by histogram. Data shown are mean ± SD of triplicate measurements that have been repeated three times with similar results. * *p* < 0.05, ** *p* < 0.01, ns = not significant.

To determine the role of SIRT2 in HFCS-induced hepatic steatosis, we successfully obtained SIRT2 KO mice based on Clustered Regularly Interspaced Short Palindromic Repeats (CRISPR)/CRISPR-associated protein 9 (Cas9) system. SIRT2 KO mice and their wildtype littermates (SIRT2 WT) were fed with HFCS diet (HFC diet plus 10% sucrose water) for 12 weeks and then assessed for liver injury, serum metabolomics, and fecal 16S rRNA sequencing (Figure 1). Interestingly, under a normal chow diet (NCD), SIRT2 KO mice exhibited little difference in the body size and weight compared with those of SIRT2 WT mice up to 20 weeks of age (Figure S1A,B). However, the SIRT2 WT mice under HFCS diet got larger body size than those under NCD or HFD diet (Figure S1C). In order to investigate the effect of HFCS diet on SIRT2 expression, we analyzed mRNA and protein levels in liver tissue samples from mice fed with HFCS and NCD diets, respectively. Our findings indicate a notable decrease in SIRT2 expression at both protein and mRNA levels in wild-type mice which were fed with HFCS, in comparison to those which were fed with NCD (Figure S1D,E). More important, with HFCS diet for 12 weeks, SIRT2 KO mice displayed a noticeably larger body size and higher body weight than SIRT2 WT mice (Figure 2A). It should be noted that there was no significant difference in the food intake between WT and SIRT2 KO mice (Figure 2B), ruling out the possibility that the difference of body size and weight was due to food intake. Consistently, the weight of liver, adipose tissue, and liver/body weight ratio were remarkably increased in SIRT2 KO mice (Figure 2C,D). Moreover, histology analysis showed a more severe presence of hepatic steatosis and higher lipid storage in the liver of SIRT2 KO mice, characterized by increased lipid droplets (Figure 2E). These data demonstrate that SIRT2 KO mice are susceptible to HFCS-induced obesity and hepatic steatosis.

### 2.2. The Aggravated Metabolic Profile Is Observed in SIRT2 KO Mice on a HFCS Diet

**Figure 3:**
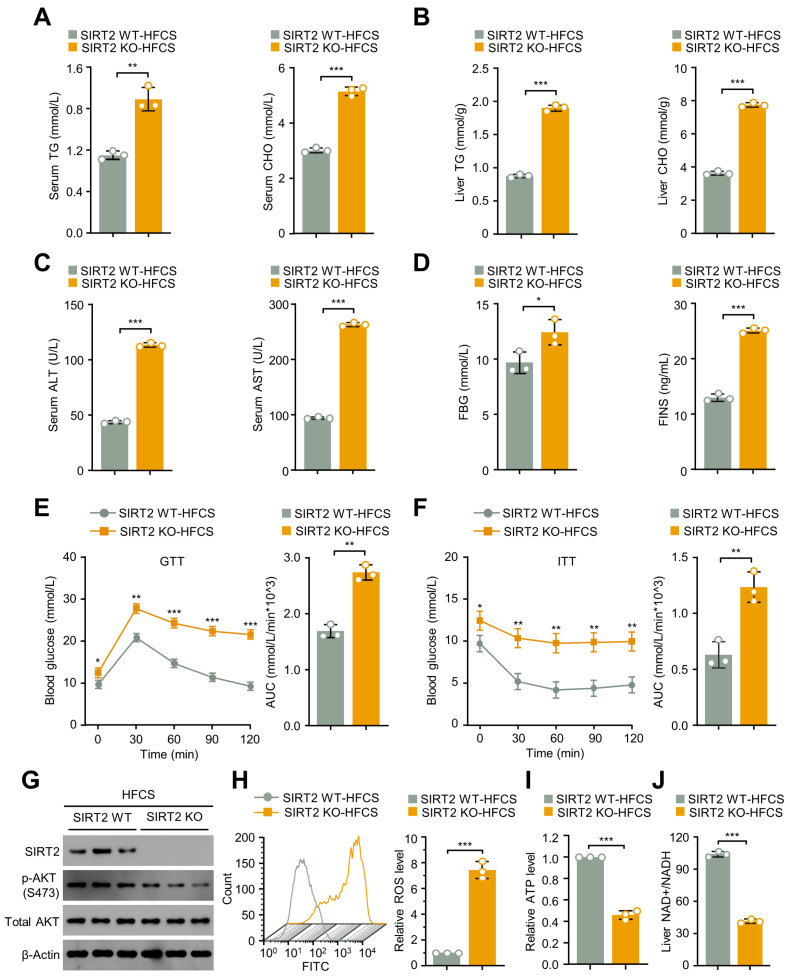
SIRT2 KO mice exhibited an attenuated metabolic profile on an HFCS diet. (**A**) Serum TG and CHO levels, (**B**) hepatic TG and CHO levels, (**C**) serum ALT and AST, (**D**) FBG and FINS levels in SIRT2 WT and KO mice fed with HFCS diet for 12 weeks (N = 9/per group). (**E**) GTT and (**F**) ITT of mice at the end of 12 weeks’ HFCS feeding. The AUC was calculated to quantitatively analyze GTT and ITT (N = 9/per group). (**G**) Western blotting analysis of SIRT2, phospho-AKT (p-AKT), and total AKT expressed in liver from SIRT2 WT and KO mice with an HFCS diet for 12 weeks. Three representative mice from each group were selected for this experiment. (**H**) ROS production in the indicated groups was analyzed using flow cytometry. (**I**) ATP production in the indicated groups was analyzed using luminometer. (**J**) The NAD^+^ to NADH ratio was analyzed in the livers of mice fed with HFCS diet for 12 weeks. Data shown are mean ± SD of triplicate measurements that have been repeated three times with similar results. * *p* < 0.05, ** *p* < 0.01, *** *p* < 0.001.

To verify if the increased hepatic lipid storage affords harmful effects on systemic energy homeostasis in SIRT2 KO mice, the circulating metabolic profile was testified. As shown in Figure 3A,B, the concentrations of triglyceride (TG) and cholesterol (CHO) in both fasting serum and liver were significantly higher in SIRT2 KO mice than in WT mice after 12 weeks of HFCS. Combined with the results that serum alanine aminotransferase (ALT) and aspartate aminotransferase (AST) levels were significantly elevated in SIRT2 KO mice fed with HFCS compared to those of WT mice (Figure 3C), these data collectively indicate an aggravated severity of liver dysfunction in SIRT2 KO mice. Regarding glucose metabolism, the levels of fasting blood glucose (FBG) and fasting serum insulin (FINS) were increased in SIRT2 KO mice with an HFCS diet (Figure 3D). A glucose tolerance test (GTT) and an insulin tolerance test (ITT) indicated that SIRT2 KO HFCS mice displayed less efficient clearance of serum glucose than WT HFCS mice, as evidenced by the increased area under the curve (AUC) of both GTT and ITT, respectively (Figure 3E,F). Subsequently, the response sensitivity of insulin in SIRT2 KO and WT was estimated in the liver tissues by Western blotting analysis of insulin receptor signaling transduction as previously described in [18]. As expected, the phosphorylation of Akt (S473) was significantly decreased in the liver tissues of the SIRT2 KO HFCS mice (Figure 3G), indicating the weakened glucose and insulin tolerance of SIRT2 KO HFCS mice. Previous research has demonstrated that reactive oxygen species (ROS) can cause lipid peroxidation, resulting in inflammation and liver damage. Fatty livers are particularly vulnerable to increased ROS and oxidative stress, which can lead to mitochondrial dysfunction, decreased levels of hepatocyte antioxidants, inflammation, and ultimately NASH and fibrosis. Therefore, we investigated whether SIRT2 KO mice fed with an HFCS diet experienced greater oxidative stress-related liver injury compared to their WT littermates. Consistent with our hypothesis, the liver tissues of SIRT2 KO mice fed with HFCS exhibited significantly elevated ROS levels (Figure 3H), along with decreased ATP levels (Figure 3I), indicating exacerbated mitochondrial dysfunction. Nicotinamide adenine dinucleotide (NAD^+^) is a coenzyme of various dehydrogenase/oxidase enzymes in organisms. Upon receiving hydrogen and electrons removed by oxidative substances, NAD^+^ is converted into reduced nicotinamide adenine dinucleotide (NADH). Hence, the NAD^+^/NADH ratio reflects the oxidative stress status. The liver tissues of SIRT2 KO mice fed with HFCS diet showed a significantly decreased NAD^+^/NADH ratio compared to their WT littermates (Figure 3J), indicating a more severe hepatic oxidative status in SIRT2 KO mice. These findings collectively suggest that SIRT2 deficiency accelerates NAFLD-NASH progression by causing more severe oxidative stress injury and mitochondrial dysfunction in the liver.

### 2.3. Severe NASH and Fibrosis Occur in SIRT2 KO Mice Fed with HFCS Diet for 12 Weeks

**Figure 4:**
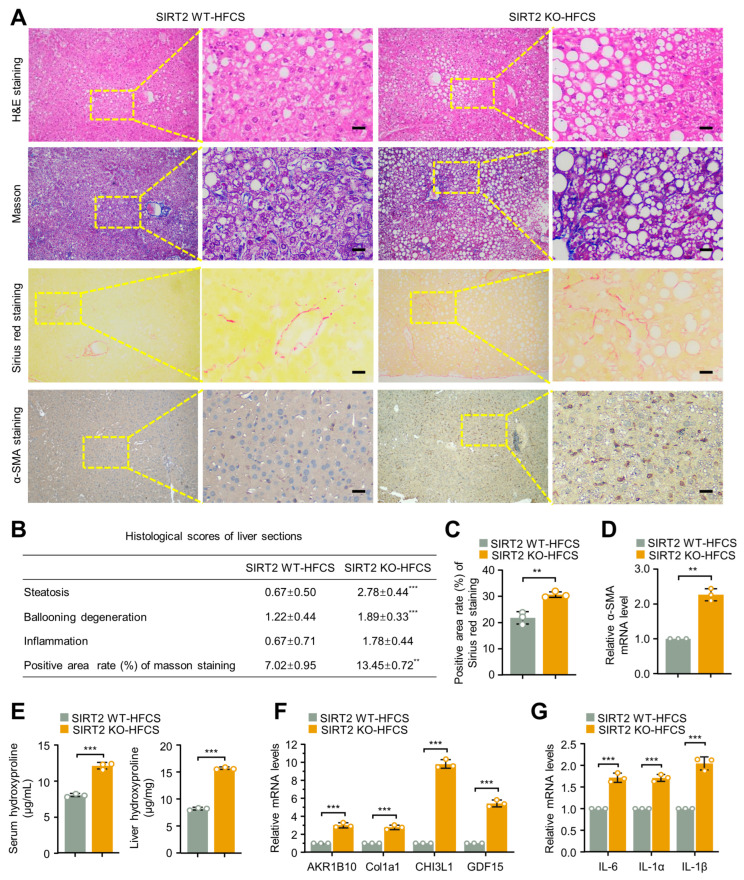
SIRT2 deficiency caused serious liver steatosis, inflammation, and fibrosis in mice with HFCS diet. (**A**) Representative H&E staining, Masson staining, Sirius red staining, and immunohistochemistry (IHC) staining of α-SMA images of liver tissues from SIRT2 WT and KO mice with HFCS diet for 12 weeks (scale bar, 50 μm). (**B**) NAFLD activity scores were assessed using the NASH Clinical Research Network (CRN) scoring system; and histological scores of liver steatosis, ballooning degeneration, inflammation, and Masson staining were calculated. (**C**) Positive area rate of Sirius Red staining of liver sections was evaluated in SIRT2 WT and KO mice with an HFCS diet for 12 weeks. (**D**) The mRNA levels of α-SMA of liver tissues from HFCS-feeding mice were analyzed by qRT-PCR (N = 9/per group). (**E**) Serum and hepatic HYP levels were analyzed in HFCS-feeding mice (N = 9/per group). (**F**) The mRNA levels of hepatic fibrosis-related genes AKR1B10, Col1a1, CHI3L1, and GDF15 were analyzed in the liver from SIRT2 WT and KO mice with an HFCS diet for 12 weeks (N = 9/per group). (**G**) The mRNA levels of inflammatory factors IL-6, IL-1α, and IL-1β were analyzed in the liver of SIRT2 WT and KO mice with an HFCS diet for 12 weeks (N = 9/per group). Data shown are mean ± SD of triplicate measurements that have been repeated three times with similar results. ** *p* < 0.01, *** *p* < 0.001.

Since we previously observed that SIRT2 deficiency significantly aggravated HFCS-induced obesity and hepatic lipid storage, we next examined in more detail of the liver sections of SIRT2 KO and SIRT2 WT mice. A histological assay revealed a much more severe presence of steatohepatitis in SIRT2 KO mice fed with HFCS diet characterized by an increased lipidosis and hepatocyte ballooning degeneration in liver tissues (Figure 4A,B). SIRT2 KO mice with HFCS diet also demonstrated more severe fibrosis with significantly increased collagen distribution areas as stained by Masson and Sirius red (Figure 4A–C), more activated hepatic stellate cells evidenced by increased alpha-smooth muscle actin (α-SMA) mRNA and protein levels (Figure 4A,D), and higher collagen levels evidenced by higher serum and liver hydroxyproline (HYP) levels (Figure 4E). Moreover, several crucial fibrosis-inducing genes including aldo-keto reductase family 1, member B10 (AKR1B10), collagentypeIalpha1 (Col1a1), chitinase-3-like protein 1 (CHI3L1), and growth differentiation factor 15 (GDF15) were significantly upregulated in the liver tissues of SIRT2 KO HFCS-fed mice as indicated by qRT-PCR analysis (Figure 4F). Furthermore, expressions of hepatic proinflammatory cytokines including IL-6, IL-1α, and IL-1β were increased in SIRT2 KO mice fed with HFCS as measured by qRT-PCR, indicating an aggravated inflammatory reaction under SIRT2 deficiency (Figure 4G). Collectively, these findings indicate that more severe NASH and fibrosis are formed in the liver tissues of HFCS-fed diet SIRT2 KO mice, demonstrating an accelerating role of SIRT2 deficiency in the process of NAFLD-NASH progression.

### 2.4. SIRT2 Deficiency Promotes Lipid Deposition and Inflammation In Vitro Cultured Cells

**Figure 5:**
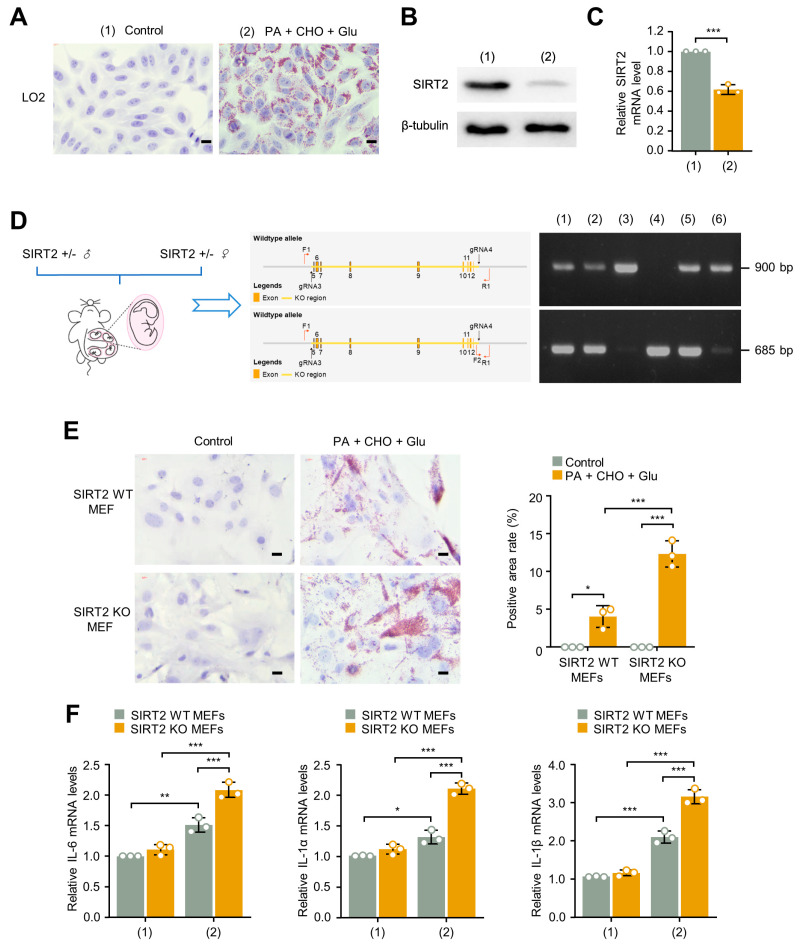
SIRT2 deficiency promotes lipid deposition and inflammation under palmitic acid (PA), cholesterol (CHO), and glucose (Glu) conditions in vitro cultured cells. The LO2 cells were treated with control or PA + CHO + Glu solutions, respectively. (**A**) The TG deposition was assessed by Oil Red O staining (scale bar, 50 μm), and (**B**,**C**) the SIRT2 protein and mRNA levels were measured by Western blotting and qRT-PCR, respectively, in the treated LO2 cells. (**D**) SIRT2 WT and SIRT2 KO MEFs were isolated. Genotype was identified by mouse direct PCR kit with 685 and 900 bp products representing WT and KO homozygotes, respectively. SIRT2 WT and SIRT2 KO MEFs were treated with control or PA + CHO + Glu solution, respectively. (**E**) The TG deposition and the (**F**) mRNA expression levels of inflammatory factors IL−6, IL−1α, and IL−1β were analyzed in the treated SIRT2 WT and KO MEFs (scale bar, 50 μm). * *p* < 0.05, ** *p* < 0.01, *** *p* < 0.001.

In cultured human normal immortalized hepatocyte cell line LO2, simultaneous treatment of palmitic acid (PA), cholesterol (CHO), and glucose (Glu) (PA + CHO + Glu) could induce TG accumulation (Figure 5A), accompanied by decreased SIRT2 protein and mRNA levels (Figure 5B,C). To further investigate the effect of SIRT2 deficiency in vitro, SIRT2 KO mouse embryo fibroblasts (MEFs) and SIRT2 WT MEFs were isolated and testified (Figure 5D). Treatment of PA + CHO + Glu induced TG accumulation in both SIRT2 WT and SIRT2 KO MEFs, and more importantly, SIRT2 KO MEFs exhibited more severe TG accumulation (Figure 5E). Furthermore, SIRT2 KO MEFs also exhibited higher mRNA level of inflammatory cytokines under PA + CHO + Glu treatment, indicating more intense inflammation activity under SIRT2 deficiency (Figure 5F). These results demonstrate that SIRT2 deficiency promotes lipid deposition and inflammation under PA + CHO + Glu conditions in cultured cells.

### 2.5. SIRT2 Is Downregulated in the Exacerbated Progression of Normal Liver Status to NAFLD to NASH in Clinical Patients

**Figure 6:**
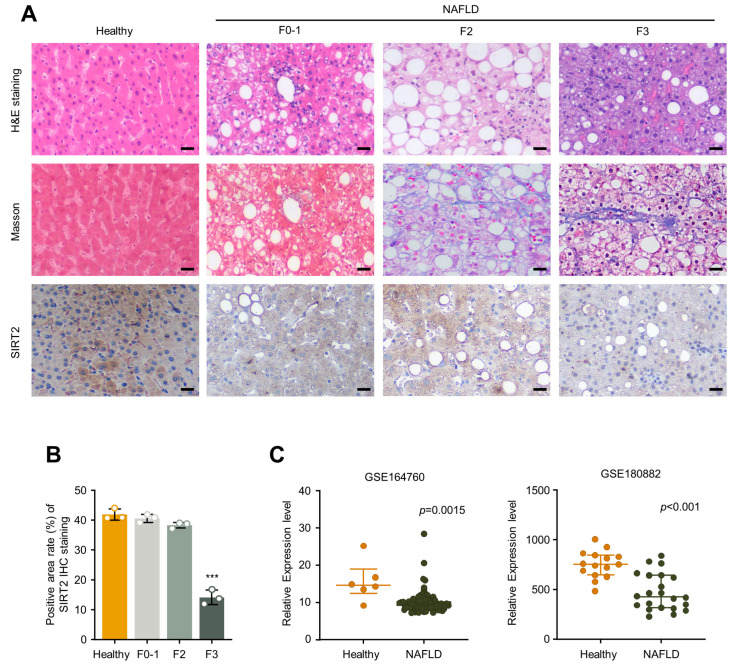
SIRT2 is downregulated in exacerbating NASH status of clinical patients. (**A**) Representative H&E staining, Masson staining, and IHC staining of SIRT2 images of hepatic sections from clinical samples (scale bar, 50 μm). (**B**) The positive area of SIRT2 IHC staining was quantified using Image J (version 1.53a). (**C**) Scatter diagrams illustrating the levels of SIRT2 in healthy and NAFLD subjects from GEO datasets GSE164760 and GSE180882. *** *p* < 0.001.

To determine the roles of SIRT2 expression in human liver fibrosis, we examined liver specimens from four stages, from healthy to NASH progression: healthy controls, patients with mild fibrosis (F0, F1), moderate fibrosis (F2), and advanced fibrosis (F3). In the indicated stages of NASH progression, we observed a decreased level of SIRT2 in advanced fibrosis (F3) compared to earlier stages of fibrosis as well as healthy controls (Figure 6A,B). To further investigate the association of SIRT2 expression and NAFLD progression in clinical NAFLD patients, we assessed the levels of SIRT2 between healthy and NAFLD human subjects using two public microarray datasets, GSE164760 and GSE180882, from the Gene Expression Omnibus (GEO) database. Interestingly, similar to the observations in mouse-derived tissue sample from our experiment, SIRT2 expression level was significantly decreased in NAFLD patients when compared with the healthy group in both datasets (Figure 6C). In summary, these data suggest that SIRT2 is critical for the disease progression from normal liver status to NAFLD to NASH in clinical patients.

### 2.6. SIRT2 Deficiency Promotes NAFLD Progression by Inducing Metabolites Alteration

**Figure 7:**
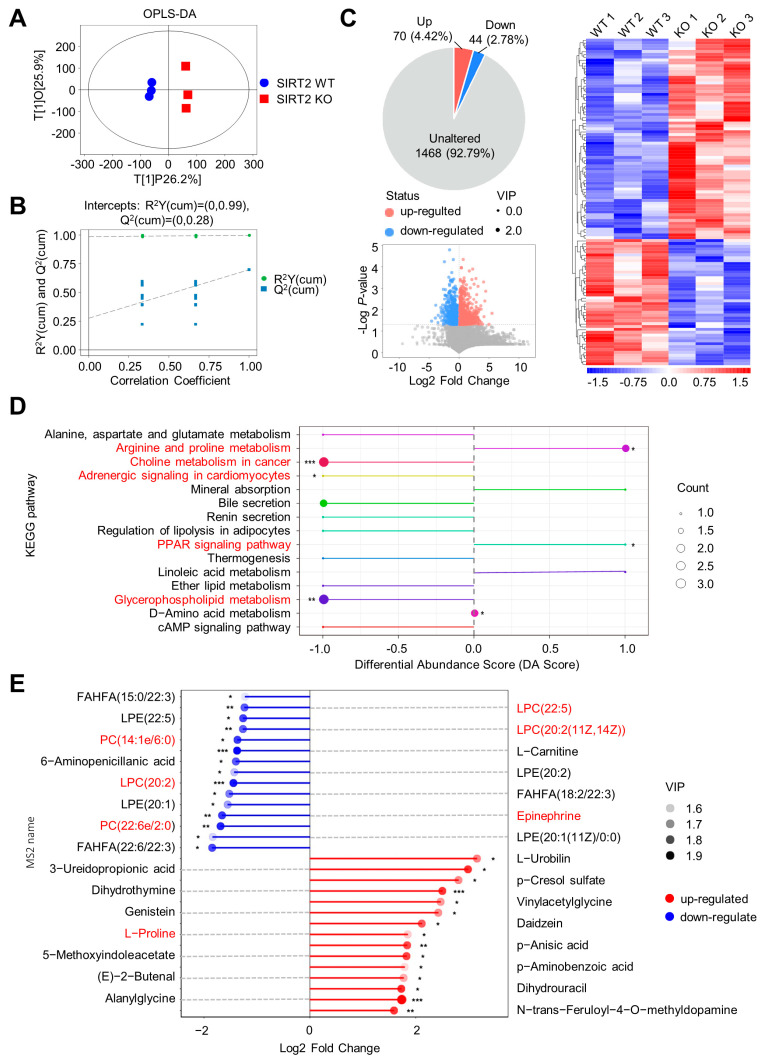
SIRT2 deficiency modulated the serum metabolites in HFCS-feeding mice. (**A**) Two−dimensional OPLS−DA score plot for the SIRT2 KO and SIRT2 WT mice (N = 3/per group). (**B**) OPLS−DA permutation test for the SIRT2 KO and SIRT2 WT mice (N = 3/per group). (**C**) The differential metabolites between the SIRT2 KO and SIRT2 WT mice were plotted in a pie chart, volcano plot, and heatmap of hierarchical clustering analysis (N = 3/per group). (**D**) KEGG pathway analysis according to all of the differential metabolites (N = 3/per group). (**E**) The top 15 up-regulated and down-regulated metabolites in SIRT2 KO mice were analyzed by matchstick analysis (N = 3/per group). * *p* < 0.05, ** *p* < 0.01, *** *p* < 0.001.

To reveal the metabolic phenotypes of NAFLD in SIRT2 KO mice that may be involved in HFCS diet induction, we performed serum metabolic analysis from HFCS-fed SIRT2 WT and SIRT2 KO mice. Serum metabolites were significantly different between SIRT2 WT and SIRT2 KO according to Orthogonal Projection Discriminant Analysis (OPLS-DA) score (Figure 7A and Figure S2A,D). The permutation test proved the applicability of this analysis model (Figure 7B and Figure S2B,E). In comparison with the SIRT2 WT mice, the SIRT2 KO mice displayed 70 up-regulated metabolites and 44 down-regulated metabolites (Figure 7C and Fiugre S2C,F and Supplementary Table S1). Kyoto Encyclopedia of Genes and Genomes (KEGG) pathway analysis showed that these differential metabolites mainly enriched into 15 pathways, of which arginine and proline metabolism, choline metabolism, adrenergic signaling, PPAR signaling, and glycerophospholipid metabolism were significantly altered (Figure 7D and Figure S2G). It was noted that among the top 15 up-regulated and down-regulated metabolites, several metabolites were enriched in the significantly changed KEGG pathways (Figure 7E and Supplementary Table S2). Generally, lysophosphatidylcholine (LPC) and phosphatidylcholines (PC), downregulated in SIRT2 KO mice and enriched in the hepatoprotective choline metabolism and glycerophospholipid metabolism pathways, inhibit the progression of NAFLD via lipotropic action and alleviating hypercholesterolemia [19,20,21]. In addition, the downregulated epinephrine was enriched in the adrenergic signaling pathway which inhibits NAFLD through the catabolism of lipid energy [22]. On the other hand, L-proline, upregulated in SIRT2-KO mice and enriched in the arginine and proline metabolism pathway, promotes the progression of NAFLD by activating immune response [23]. In summary, these results imply that SIRT2 deficiency aggravated the HFCS-induced metabolic disorder to promote the progression of NAFLD.

### 2.7. SIRT2 Deficiency Promotes Gut Microbiota Dysbiosis of HFCS-Fed Mice in the NAFLD Progression

**Figure 8:**
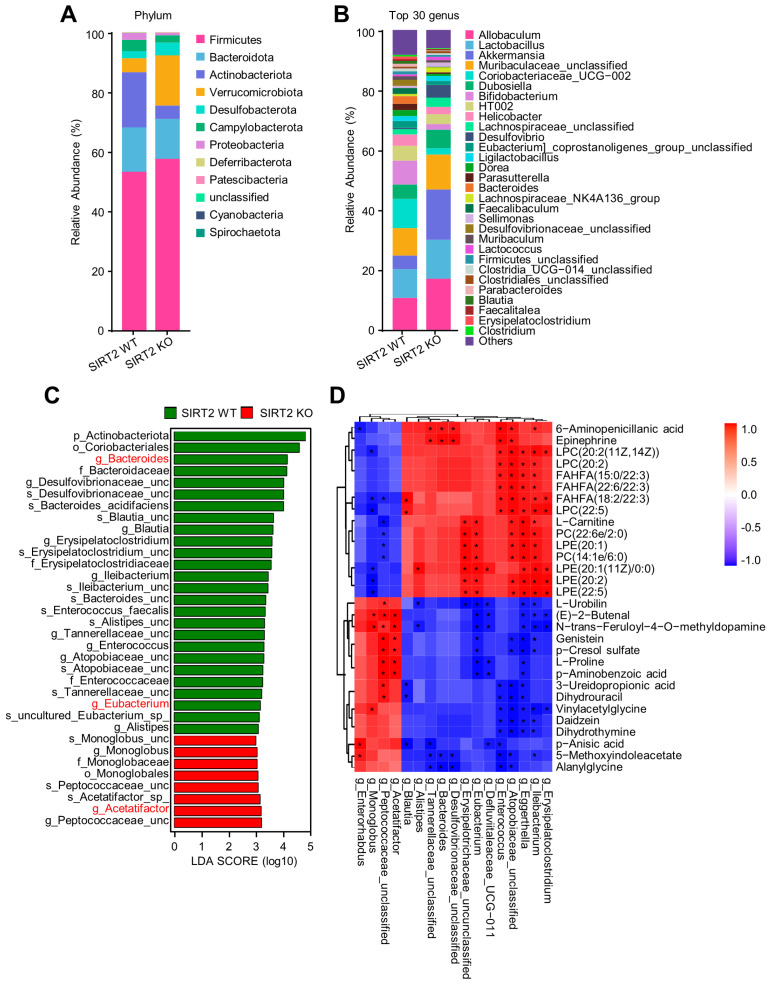
SIRT2 deficiency modulated gut microbiota dysbiosis in HFCS−feeding mice. (**A**) Relative abundance of gut microbiota at the phylum level in SIRT2 KO and WT mice (N = 3/per group). (**B**) The top 30 gut microbiota at the genus level in SIRT2 KO and WT mice. (**C**) LEfSe analysis at different taxonomy levels between the SIRT2 KO and SIRT2 WT mice. The “_unc” stands for “_unclassified”. (**D**) Correlation analysis between the differential serum metabolites and the differential gut microbiota at the genus level. * *p* < 0.05.

To explore whether gut microbiota mediates SIRT2 deficiency-induced NAFLD, fecal 16S rRNA gene sequencing was performed on HFCS-fed mice for 12 weeks. The Shannon and Chao1 indices were remarkably lower for the SIRT2 KO mice, indicating that the richness and multiplicity of the gut microbiota were significantly reduced with SIRT2 deficiency (Figure S3A). The composition of the intestinal microbiota also exhibited obvious discrimination between the SIRT2 KO and WT mice as illustrated by principal coordinate analysis (PCoA) (Figure S3B). The differential abundance of gut microbiota was clearly displayed at phylum and genus levels (Figure 8A,B and Figure S3C,D and Supplementary Tables S3 and S4). Moreover, linear discriminant analysis effect size (LEfSe) analysis demonstrated that SIRT2 regulates specific gut microbiota at different taxonomic levels (Figure 8C and Figure S4). Specifically, *Acetatifactor*, which was comprised in the core microbiome related to NAFLD [24], was more abundant in SIRT2 KO mice. Whereas, *Bacteroides* and *Eubacterium*, both probiotic bacteria [25], were decreased in SIRT2 KO mice (Figure 8C). 

Gut microbiome exists in a complex consortium of ecological and metabolic interactions [26]. The microorganism association and interaction are critical to maintain the homeostasis of gut microbiota which ultimately influence host digestion, immunity, and metabolism [27]. Generally, among the microbial community, several microorganisms will play critical roles in the initiation and progression of diseases and are usually called driver microorganisms [28]. In our study, the correlation among all the microbial genera were subsequently analyzed (Figure S5). *Bacteroides* and *Desulfovibrionaceae* positively correlated with each other. Besides this, *Eubacterium* was positively correlated with *Faecalitalea* and *Sellimonas*. This implied that the downregulation of *Bacteroides* and *Eubacterium* in SIRT2 KO mice might be a critical driver factor that induced gut microbiota dysbiosis associated with NAFLD.

Based on the above findings, the relationships between 17 differential microbial genera and 30 differential metabolites were further assessed (Figure 8D). Regarding the inhibition of NAFLD, metabolites (LPC and PC) in the downregulated choline metabolism and glycerophospholipid metabolism pathways positively correlated with a cluster of gut microbiota (*Erysipelatoclostridium, Ileibacterium, Eggerthella, Atopobiaceae_unclassified, Enterococcus, Eubacterium, Erysipelatoclostridium_unclassified*, and *Blautia*) which were downregulated in SIRT2 KO mice, and negatively correlated with two microbial genera (*Monoglobus, Peptococcaceae_unclassified*) which were upregulated in SIRT2 KO mice. In addition, epinephrine in the downregulated adrenergic signaling pathway positively correlated with a cluster of gut microbiota (*Atopobiaceae_unclassified, Enterococcus, Desulfovibrionaceae_unclassified, Bacteroides,* and *Tannerellaceae_unclassified*) which were downregulated in SIRT2 KO mice. As to the promotion of NAFLD, L-proline, which was enriched in the upregulated arginine and proline metabolism pathway was positively correlated with two microbial genera (*Acetatifactor* and *Peptococcaceae_unclassified*) that were upregulated in SIRT2 KO mice. Collectively, these results demonstrated that there is an intensive connection between the serum metabolic disorder and the gut microbiota dysbiosis caused by SIRT2 deficiency, the interplay of which may contribute to NAFLD progression.

### 2.8. Correlation Analysis among Gut Microbiota, Metabolism, and Mice Phenotype

**Figure 9:**
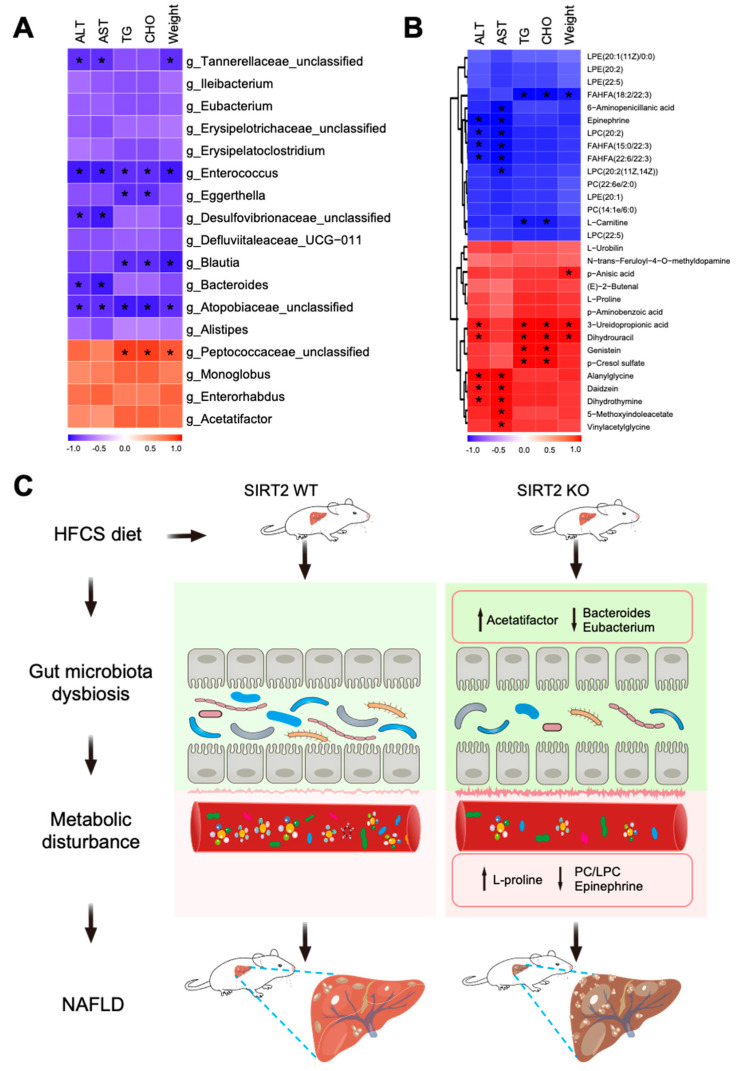
Correlation among gut metabolism, serum metabolites, and mouse phenotype. (**A**) Spearman’s correlation analysis between the differential gut microbiota at the genus level and the mouse phenotype. (**B**) Spearman’s correlation analysis between the differential serum metabolites and the mouse phenotype. The correlation analysis value is represented by the colors of the grinds. (**C**) A proposed model depicting the regulatory mechanism of SIRT2 deficiency in the progression of NAFLD. * *p* < 0.05.

In order to further explore the pathogenesis of intestinal microbiota dysbiosis and serum metabolic disorder in contributing to HFCS-induced hepatic steatosis, correlations between mice phenotype with gut microbiota and serum metabolites were identified. Several microbes (*Tannerellaceae_unclassified*, *Enterococcus*, *Eggerthella, Desulfovibrionaceae_unclassified*, *Blautia*, *Bacteroides*, and *Atopobiaceae_unclassified*) that depleted in SIRT2 KO mice exhibited a negative correlation with the NAFLD phenotypes of mice. Among them, *Enterococcus* and *Atopobiaceae_unclassified* seemed to play more important roles with being correlated with multiple indicators of NAFLD (Figure 9A). The metabolites depleted in SIRT2 KO mice, such as FAHFA (18:2/22:3), 6-aminopenicillanic acid, epinephrine, LPC (20:2), FAHFA (15:0/22:3), FAHFA (22:6/22:3), LPC (20:2(11Z,14Z)), and L-carnitine were negatively correlated with different NAFLD phenotypes. However, the metabolites excessive in SIRT2 KO mice (p-anisic acid, 3-ureidopropionic acid, dihydrouracil, p-cresol sulfate, alanylglycine, daidzein, dihydrothymine, 5-methoxyindoleacetate, and vinylacetylglycine) were positively correlated with NAFLD phenotypes (Figure 9B). Considering all these above results, we proposed an assumption that SIRT2 KO mice suffered from more serious gut microbiota dysbiosis and metabolic disorder induced by HFCS diet, which might account for the promotion of the NAFLD progression characterized by larger body weight and higher serum ALT, AST, TG, and CHO levels (Figure 9C).

## 3. Discussion

The global issue of NAFLD is strongly linked to dietary patterns [20,21,22]. Research has identified dietary cholesterol as a major lipotoxic molecule that triggers and spreads sterile inflammation leading to fibrosis, and advancing the progression of NAFLD [23]. A meta-analysis has demonstrated that consuming high amounts of liquid sugar significantly raises intrahepatocellular lipid levels and ALT levels [24]. In a previous study, mice were injected with adenovirus overexpressing SIRT2 and fed a high-fat and high-glucose diet for 12 weeks. Both the OE (overexpression)-SIRT2 group and WT groups of mice developed NAFLD. However, H&E staining of liver tissue and biochemical assays showed that the OE-SIRT2 group had reduced severity of steatosis, lower levels of triglyceride and ALT, and decreased inflammatory indicators compared to the WT group. No significant liver fibrosis was observed during this induction period [16]. These findings suggest that OE-SIRT2 alleviates liver steatosis and inflammation during NAFLD progression induced by a high-fat and high-sugar diet, without affecting the fibrosis status of the liver within the given induction time. In another study, SIRT KO and WT mice were fed with a high-fat diet for several weeks. Liver steatosis and mRNA levels of inflammatory genes were measured, but little difference in mRNA expression was observed. In our study, we fed SIRT KO and WT mice with a combination of high-fat, high-cholesterol, and high-sugar diet (HFCS) for 12 weeks. We observed not only steatosis and higher biochemical indicator phenotypic changes, but also fibrosis phenotypes in the liver of mice fed with HFCS. This suggests that sugar, fat, and cholesterol have a synergistic effect on exacerbating the NAFLD process in a much shorter period of time.

Studies have shown that gender plays a role in the onset and severity of NAFLD. Men are at a higher risk of developing NAFLD than women, while women who do develop the disease are more likely to progress to advanced liver fibrosis [25,26,27]. Age is also a significant factor in NAFLD progression, as menopausal women over 50 are particularly susceptible to advanced liver fibrosis [28]. Our study did not find a significant difference between male and female mice, which might be due to the limited duration of diet induction (12 weeks) and the age of the mice (less than 20 weeks). It has been previously published that SIRT2 protected against metabolic disorders, oxidative stress, mitochondrial dysfunction, insulin resistance, inflammation, obesity, and cardiomyocyte senescence [29]. Our study found that SIRT2 deficiency exacerbated hepatic steatosis, inflammation, and fibrosis, while also impairing insulin sensitivity and aggravating obesity. These findings suggest that SIRT2 deficiency plays a role in promoting diet-induced NASH and metabolic dysfunction. 

NAFLD is a chronic disease associated with metabolic dysfunction [5]. The accumulation of lipids in the liver stimulates the release of pro-inflammatory cytokines from Kupffer cells, which can activate the inflammatory response, and finally drive the progression of NAFLD [30]. In this study, we identified four dominantly differential metabolites enriched in 4 KEGG pathways, which play various NAFLD-related physiological roles, including lipid metabolism, collagen synthesis, insulin sensitivity, as well as inflammation. In light of previous studies, choline metabolism and adrenergic signaling pathway performed hepatoprotective effects through lipotropic action [31]. In addition, the activated glycerophospholipids metabolism pathway can effectively alleviate hypercholesterolemia and obesity-related complications [32]. Dysfunction of choline metabolism, adrenergic signaling, and glycerophospholipid metabolism could lead to a range of metabolism-related diseases, including NASH [33]. Specifically speaking, the differential metabolites enriched in these hepatoprotective pathways performed NAFLD-inhibition functions. PC could enhance cholesterol oxidation, block fatty acid synthesis, and relieve hepatic lipid deposition [34]. PC, together with its metabolic product LPC, was reported to be declined in NAFLD patients [35]. As a stress hormone, epinephrine can activate adrenergic receptor β3 (ADR3) in the liver, which inhibits lipogenesis, promotes lipolysis, and stimulates hepatic glucose release [36]. On the other hand, the arginine and proline metabolism pathway can activate immune response [37], which is a critical factor that triggers NAFLD progression. The differential metabolite, L-proline, enriched in this harmful pathway could exert NAFLD promoting effect. To be more specific, L-proline is a major amino acid included in hepatic collagen synthesis, and elevated serum L-proline level indicates hepatic fibrosis [38]. In our study, the SIRT2 KO mice exhibited significantly declined hepatoprotective metabolites (PC, LPC, and epinephrine) and elevated harmful metabolites (l-proline), demonstrating that SIRT2 deficiency may promote NAFLD progression through metabolic disorders.

Intestinal microbiota is the microbial community in the gastrointestinal tract that plays a critical physiological role in host digestion, immunity, and metabolism [39]. There is increasing evidence that dysbiosis of gut microbiota can influence a variety of pathologic conditions, including NAFLD [40]. Our study found that gut microbiota mediates HFCS-induced NAFLD in SIRT2 KO mice. *Bacteroides* and *Eubacterium* were significantly reduced in SIRT2 KO mice, along with bacterial richness. Studies have found that *Bacteroides* can lower cholesterol levels, prevent obesity, and improve insulin sensitivity [4,41]. Clinical data also showed that *Bacteroides* were negatively associated with total serum CHO in human hypercholesteremia patients [4], and depletion of *Bacteroides* has been demonstrated in human NASH patients [42]. Besides this, *Eubacterium* can improve hepatic lipid deposition by producing butyric acid, regulating bile acid metabolism, and increasing the activity of fatty acid oxidation genes in the liver, all of which contribute to a reduction in NAFLD [43]. On the other hand, consistent with previous studies that *Acetatifactor* was included in the core microbiome related to NAFLD [44], our study also showed that *Acetatifactor* exhibited a higher abundance in the SIRT2 KO mice. The SIRT2 protein belongs to the sirtuin family, a conserved group of NAD^+^-dependent deacetylases that play a vital role in regulating cellular metabolism. By deacetylating key enzymes involved in glycolysis, the tricarboxylic acid cycle, fatty acid oxidation, and glutaminolysis, these proteins help maintain metabolic homeostasis [45,46,47]. Thus, deficiency of SIRT2 may lead to metabolic abnormalities of the targeted cells. Previous studies have shown that other members of the sirtuin family, such as SIRT3 and SIRT1, are also involved in maintaining gut microbiota balance by protecting intestinal barrier integrity and promoting antimicrobial peptide production [48,49]. SIRT2, specifically, has been found to help maintain intestinal cell homeostasis by preventing inflammatory processes [50,51]. Based on this information, it is possible that SIRT2 deficiency may disrupt gut microbiota balance by compromising the intestinal barrier and altering the production of antimicrobial metabolites. However, additional research is still needed to investigate this hypothesis in-depth. Taken together, SIRT2 deficiency promoted NAFLD progression through a decreased abundance of probiotic gut microbes and an increased abundance of harmful gut microbes. 

Besides all this, increasing evidence suggests that the gut microbiota significantly influences the levels of metabolites in host blood [52]. The obesity-inhibition metabolite PC was proved to be positively correlated with beneficial *Bifidobacterium*, *Allobaculum*, and *Anaerostipes* [53]. The serum metabolite epinephrine is closely linked with probiotic *Bacteroides*, and both of them performed functions of mobilization and catabolism of lipid energy [54]. In our study, under the HFCS diet, remarkable positive correlations between the gut microbiota dysbiosis and metabolic disorder were explored in the SIRT2 KO mice, revealing that SIRT2 deficiency may promote NAFLD progression through the collaboration between metabolites and gut microbiota.

Our study revealed that SIRT2 is crucial for the progression of NAFLD, which is related to metabolic disorders and gut microbiota dysbiosis. We showed that the SIRT2 KO mice were more susceptible to HFCS-induced obesity and hepatic steatosis. Under HFCS diet, SIRT2 KO mice exhibited an aggravated metabolic profile and promoted NAFLD-NASH progression both in vitro and in vivo. Mechanically, SIRT2 deficiency induced serum metabolite alteration and is associated with gut microbiota dysbiosis. In clinical NAFLD patients, SIRT2 was down-regulated and negatively correlated with the healthy-NAFLD-NASH progression. SIRT2 deficiency accelerates HFCS-induced NAFLD-NASH progression by inducing alteration of gut microbiota and changes of metabolites.

## 4. Materials and Methods

### 4.1. Generation of SIRT2 Knockout Mice

SIRT2 globally knockout mice on a C57BL/6 background were generated using CRISPR/Cas9 method (Guangzhou Cyagen Biosciences Inc., Guangzhou, China). Briefly, C57BL/6 eggs were microinjected with plasmids encoded with Cas9 and SIRT2 guide RNAs, and transgenic embryos were subsequently grafted into pseudopregnant mice. SIRT2 homozygote mice were obtained by crossing male and female SIRT2 heterozygote mice. For mouse embryo genotype identification, genomic DNA was prepared from the tail tips of 14-day-old embryos and the SIRT2 mutation was identified by twice PCR amplification. The genotyping primers for homozygotes are 5′-TGCCCTCAAAGGTCAGAGGCACAG-3′ (forward primer) and 5′-CCAAAACTAATAACCGTCTTCGTC-3′ (reverse primer). The genotyping primers for wildtype are 5′-CTGTCATTCTAGACTCCACTGAGC-3′ (forward primer) and 5′-CCAAAACTAATAACCGTCTTCGTC-3′ (reverse primer).

### 4.2. Animal Care

Male SIRT2 KO mice (6–8 weeks) in C57BL/6 background and their corresponding WT littermates were used in this study. It was maintained in a specific pathogen free (SPF) animal center where mice were monitored on a 12-h light-dark cycle with an ambient temperature of (22 ± 0.5 °C). All animal studies were conformed to the guidelines of the Institutional Animal Care Committee of Beijing Institute of Biotechnology. Mice were fed with either normal chow diet (NCD) or high-fat/high-cholesterol/high-sucrose [HFCS, containing 40% HFC diet (D12108C, Research Diets, Inc., New Brunswick, NJ, USA) and 10% sucrose water (S8271, Beijing Solarbio Science & Technology Co., Ltd., Beijing, China)] diet ad libitum for 12 weeks (N = 9/per group). 

### 4.3. Cell Culture and Treatment 

SIRT2 KO MEF and its corresponding WT type were isolated from the embryos at day 10–14 of the single heterozygous female SIRT2 KO mouse that had been mated to a heterozygous male mouse. After discarding the head and organs, the remaining components were minced and isolated into single cells using trypsin. We then plated and cultured the isolated MEFs in Dulbecco’s modified Eagle’s medium (DMEM) containing 10% FBS with 100 I.U. penicillin, and 100 µg/mL streptomycin in a CO_2_ incubator (37 °C, 5% CO_2_), and gestated to identify the genotype by a mouse direct PCR kit (B40013, Bimake, Houston, TX, USA). LO_2_ (immortalized epithelial cell line derived from human liver) was purchased from American Type Culture Collection (ATCC) (Manassas, VA, USA) and cultured in DMEM supplemented with 10% FBS containing 100 I.U. penicillin, and 100 µg/mL streptomycin. MEFs and LO_2_ cells were treated with palmitic acid (250 μmol/L, KT003, KunChuang Co. Ltd., Xi’an, China), cholesterol (200 μg/mL, C49541, Sigma-Aldrich, St. Louis, MO, USA), and glucose (10 mmol/L, KC05X, KunChuang Co. Ltd., Xi’an, China). 

### 4.4. Biochemical Assay

Levels of serum alanine aminotransferase (ALT), aspartate aminotransferase (AST), triglycerides (TG), and cholesterol (CHO) were determined using an automatic biochemical analyzer (7170, Hitachi, Tokyo, Japan). Liver tissue was homogenized in a standard mix and the levels of TG and CHO were assessed using a triglyceride assay kit (A110-1-1, Nanjing Jiancheng Bioengineering Institute, Nanjing, China) and a total cholesterol assay kit (A111-1-1, Nanjing Jiancheng Bioengineering Institute, Nanjing, China). After fasting overnight, blood samples were collected. Fast blood glucose (FBG) and fast serum insulin (FINS) levels were detected using glucometer (Roche, Indianapolis, IN, USA) and mouse insulin enzyme linked immunosorbent assay (ELISA) kit (90080, Crystal Chem, IL, USA), respectively. The ratio of oxidized nicotinamide adenine dinucleotide (NAD^+^)/NADH was measured using the Coenzyme ⅠNAD (H) content test kit (A114-1-4, Nanjing Jiancheng Bioengineering Institute, Nanjing, China). The hydroxyproline (HYP) concentrations of serum and liver were measured with the hydroxyproline assay kit (A030-2-1, Nanjing Jiancheng Bioengineering Institute, Nanjing, China).

### 4.5. Glucose and Insulin Tolerance Tests

Insulin tolerance test (ITT) and glucose tolerance test (GTT) were monitored after fasting for 6 h. For ITTs, mice were intraperitoneally injected with insulin (0.75 U/kg, Novolin R, Novo Nordisk, Bagsværd, Denmark). For GTTs, mice were intraperitoneally injected with glucose (1 g/kg; Sigma-Aldrich, St. Louis, MO, USA). For both ITTs and GTTs, blood was obtained from the tail vein before the injection (time point 0) and at the time points of 30, 60, 90, and 120 min after injection. Area under the curve (AUC) was calculated to reflect insulin and glucose tolerance levels. 

### 4.6. ROS Assay

To measure the ROS level of hepatic tissue, the ROS probe 2′,7′-Dichlorofluorescin diacetate (DCFH-DA, Nanjing Jiancheng Bioengineering Institute, Nanjing, China) was utilized following the manufacturer’s instruction [55,56]. Single-cell suspension of liver tissue was obtained by grinding and then filtering through a 70-µM cell strainer, which was treated with 10 μmol/L DCFH-DA probe and incubated in the dark at 37 °C for 30 min. Next, the fluorescence intensity was determined at Ex/Em 488/525 nm using flow cytometry. The data were analyzed by Flow Jo (version 10.4).

### 4.7. ATP Assay

To measure the ATP concentrations of hepatic tissues, the ATP Colorimetric Assay kit (Biyuntian, Wuxi, China) was used to determine ATP production, according to the manufacturer’s protocols. The mouse liver tissue was crushed and homogenized in the RIPA lysate containing 1 mM PMSF, completely lysed and boiled for 5 min before analysis. Luminescence was read on a TD-20/20 Luminometer (Turner Designs, Sunnyvale, CA, USA), and values were calculated based on an ATP standard curve. 

### 4.8. Immunohistochemistry (IHC) and Histological Analyses

Liver tissues were fixed with 4% paraformaldehyde and embedded by paraffin. The samples were sliced into 5 μm sections and were stained with hematoxylin and eosin (H&E), Masson, and Sirius Red according to standard protocol [57]. Sirius Red and Masson staining were used to assess the degree of fibrosis, and the positive staining area was quantified by Image J (version 1.53a). Steatosis, hepatocyte ballooning, and lobular inflammation were evaluated according to the NASH-Clinical Research Network (CRN) criteria [58]. 

For IHC staining, slides were autoclaved with sodium citrate for antigen repair, followed by applying 1% hydrogen peroxide to inactivate the endogenous peroxidase and blocking with goat serum (2%). Slides were incubated with primary antibodies including α-SMA (Proteintech, Wuhan, China, Cat No. 23660-1-AP, 1:50) and SIRT2 (Proteintech, Wuhan, China, Cat No. 19655-1-AP, 1:100) at 4 °C overnight. The signal was detected with DAB (Millipore, Burlington, MA, USA).

### 4.9. Oil Red O Staining

To analyze the lipid composition of liver tissue and cells, Oil Red O staining was performed. In preparation, fresh liver tissues were embedded in optimal cutting temperature (OCT) compound and frozen in liquid nitrogen to obtain frozen sections. As to cell samples, a phosphate-buffered saline (PBS) was used to wash cells, and 4% paraformaldehyde was used as a fixative for 30 min at room temperature. Then liver frozen sections and cells were stained with Oil Red O (RK006, Report Biotech, Hebei, China) for 30 min and examined by light microscopy. Quantitative analysis was performed by Image J software (version 1.53a).

### 4.10. Total RNA Extraction and Real-Time Quantitative PCR (qRT-PCR)

Total RNA of hepatic tissue and indicated cell samples were isolated using TRIzol reagent according to the manufacturer’s protocol (Thermo Fisher Scientific, Waltham, MA, USA). RNA was reverse transcribed into cDNA by Quantscript RT Kit (Takara, Tokyo, Japan). Real-time PCR was performed with primers listed in Supplementary Table S5.

### 4.11. Metabolome Analysis

Serum metabolome profiling was examined using an ultra-high performance liquid chromatography system (UHPLC, Thermo Fisher Scientific) and an Orbitrap Exploris 120 mass spectrometer (OE MS, Thermo Fisher Scientific) (N = 3/per group). The quadrupole electrostatic field Orbitrap mass spectrometer was utilized to obtain informative MS/MS spectra during LC/MS experiments. ProteoWizard was used for converting raw data files obtained from MS into mzXML format. An in-house program (an automated data analysis developed by R, Biotree, Shanghai, China) was used to process the detection of peaks, data extraction, and data alignment, followed by integration. In this work, metabolites were identified by the positive and negative ionization modes. The in-house MS2 database was applied to identify the metabolites. OPLS-DA was carried out to visualize the distribution and the grouping of the samples. The value of variable importance in the projection (VIP) of the first principal component in OPLS-DA analysis was obtained. The metabolites with VIP > 1 and *p* < 0.05 (student *t*-test) were considered as significantly changed metabolites.

### 4.12. Analysis of Gut Microbiota

To evaluate the alterations of gut microbiota caused by the SIRT2 knockout, DNA of fecal samples were extracted using Hipure soil DNA kit (Magen, Guangzhou, China) (N = 3/per group). The 16S rRNA V3–V4 region was amplified and sequenced using MiSeq platform (Illumina, San Diego, CA, USA). Raw data were processed to obtain clean data, and then the Amplicon Sequence variants (ASVs) feature list and feature sequence were constructed by divided amplicon denoising algorithm (DADA2). Diversity and richness of the community were identified by the Chao1 index, Shannon index, and principal Coordinate Analysis (PCoA). The abundance of phylum and genus levels was shown by stacked bar chart and heatmap. LEfSe was used to highlight core phenotypes of bacteria from domain to species that contribute to microbiota composition variation. Spearman analysis was used to calculate the top 30 microbial abundance at the genus level, and a heat map with the correlation was drawn based on the correlation index and significant P values between the two dominant bacterial groups. Spearman correlation analysis was also utilized to illustrate the relationship among metabolites, intestinal microbiota, and mice phenotype, respectively.

### 4.13. Human Clinical Samples and Gene Expression Omnibus (GEO) Dataset Analysis

The clinical liver tissue samples of NAFLD patients and healthy individuals were obtained from the Third Hospital of Hebei Medical University. The approval for the clinical research protocol was obtained from the Clinical Research Ethics Committee of Hebei Medical University. Degree of liver fibrosis was graded according to the NASH CRN histologic scoring system [58]. The GSE164760 and GSE180882 datasets were downloaded from the GEO database. The GSE164760 dataset contained gene expression profiles of liver tissues from 74 NAFLD patients and 6 healthy controls. The GSE180882 dataset contained gene expression profiles of liver tissues from 22 NAFLD patients and 15 healthy controls.

### 4.14. Statistical Analysis 

All the experiments were repeated at least three times. All the quantitative data were reported as the means ± standard deviations. Mann-Whitney U or Student’s *t*-test was utilized for the comparisons between the two groups. One-way analysis of variance or the Kruskal-Wallis test was used to compare the multiple groups. Statistical analysis was performed by the SPSS software (version 26.0), and GraphPad Prism 8.0 was utilized to make graphs. *p* < 0.05 indicated statistically significant.

## 5. Conclusions

In conclusion, this study shows that a prolonged HFCS diet can induce spontaneous NAFLD in SIRT2 KO mice through modulating serum metabolism and gut microbiota. The serum metabolic disorder and the gut microbiota dysbiosis caused by SIRT2 deficiency is a critical driving force for diet-induced NAFLD, characterized by hepatic synthetic dysfunction, glycolipid metabolism disorders, insulin resistance, oxidative stress injury, liver lipid accumulation, inflammation, and liver fibrosis.

## Data Availability

The data sets used and/or analyzed during the current study are available from the corresponding author on reasonable request.

## Supplementary Materials

The supporting information can be downloaded at: https://www.mdpi.com/article/10.3390/ijms24108970/s1.
