# Supplementary material for: SIRT2 Deficiency Aggravates Diet-Induced Nonalcoholic Fatty Liver Disease through Modulating Gut Microbiota and Metabolites
Source: Int J Mol Sci. 2023 May 18;24(10):8970. doi: 10.3390/ijms24108970 (PMC10219207; doi:10.3390/ijms24108970)
Supplement: Supplementary file 1 [file ijms-24-08970-s001.zip › ijms-2325002-SI.pdf]

Supplementary Table S1. Metabolite profiling of the serum from SIRT2 KO and SIRT2 WT mice fed with HFCS by UHPLC-QTOF-MS (SIRT2 KO vs. SIRT2 WT).

Metabolites	FC	Log2(FC)	*P*-value
L-Urobilin	8.9179	3.1567	0.0248
3-Ureidopropionic acid	7.9253	2.9865	0.0149
p-Cresol sulfate	6.9978	2.8069	0.0457
Dihydrothymine	5.6652	2.5021	0.0006
Vinylacetylglycine	5.5378	2.4693	0.0413
Genistein	5.3704	2.4250	0.0299
Daidzein	4.3283	2.1138	0.0272
L-Proline	3.6006	1.8482	0.0209
p-Anisic acid	3.5743	1.8377	0.0047
5-Methoxyindoleacetate	3.5447	1.8256	0.0168
p-Aminobenzoic acid	3.4579	1.7899	0.0210
(E)-2-Butenal	3.4114	1.7704	0.0181
Dihydrouracil	3.3097	1.7267	0.0134
Alanylglycine	3.2853	1.7160	0.0002
N-trans-Feruloyl-4-O-methyldopamine	3.0096	1.5896	0.0018
1-Cyano-2-hydroxy-3-butene	3.0024	1.5861	0.0274
FAHFA (14:0/16:2)	2.7817	1.4760	0.0099
12-Hydroxydodecanoic acid	2.5132	1.3295	0.0311
PC (9:0/26:1)	2.4043	1.2656	0.0156
FAHFA (18:0/14:1)	2.3438	1.2288	0.0435
(R)-3-Hydroxy-tetradecanoic acid	2.2721	1.1840	0.0247
Verimol D	2.2368	1.1614	0.0232
Tropic acid	2.2085	1.1431	0.0374
Succinic acid	2.0676	1.0480	0.0307
Myristic acid	2.0031	1.0022	0.0022
1H-Indole-3-carboxaldehyde	1.9289	0.9478	0.0010
2,6 Dimethylheptanoyl carnitine	1.8360	0.8765	0.0184
(3beta,23E)-3-Hydroxy-27-norcycloart-23-en-25-one	1.8076	0.8540	0.0115
7-Methylguanine	1.8022	0.8498	0.0485
FAHFA (16:1/22:3)	1.7704	0.8241	0.0434
Hypogeic acid	1.7287	0.7897	0.0334
Oleoyl glycine	1.6750	0.7442	0.0331
1,11-Undecanedicarboxylic acid	1.6702	0.7401	0.0468
FAHFA (16:1/18:3)	1.6382	0.7121	0.0309
Prehumulinic acid	1.6080	0.6853	0.0440
Ercalcitriol	1.5422	0.6250	0.0398
PG (18:2/20:4)	1.5161	0.6004	0.0065
Creatinine	1.5150	0.5994	0.0097
Falcarinone	1.5073	0.5920	0.0136

Alpha-dimorphecolic acid	1.4475	0.5335	0.0133
(9S,10E,12Z,15Z)-9-Hydroxy-10,12,15-octadecatrienoic acid	1.4453	0.5314	0.0287
2-Phenylpropanal	1.4424	0.5285	0.0359
FAHFA (18:1/22:3)	1.3932	0.4784	0.0100
(2R)-2-Hydroxy-2-methylbutanenitrile	1.3304	0.4119	0.0470
8,15-DiHETE	1.3283	0.4096	0.0371
1,2,3,4-Tetrahydro-1-[1-hydroxy-3-(4-hydroxyphenyl)-2-propenyl]-7-methoxy-2,6-naphthalenediol	1.2856	0.3624	0.0095
L-Pyroglutamic acid	1.2562	0.3291	0.0102
Cyanuric acid	1.2494	0.3212	0.0150
N-Desmethyltramadol	1.2254	0.2932	0.0167
Mizolastine	1.2157	0.2818	0.0483
4-Pentenoic acid	1.1865	0.2467	0.0127
1-Methyl-2-pyrrolecarboxaldehyde	1.1742	0.2316	0.0409
2,6-Dimethylpyridine	1.1653	0.2208	0.0100
Suberylglycine	1.1573	0.2107	0.0225
5,7alpha-Dihydro-1,4,4,7a-tetramethyl-4H-indene	1.1542	0.2069	0.0344
Colubrinic acid	1.1346	0.1822	0.0257
Safrole	1.1312	0.1779	0.0353
Lithocholic acid glycine conjugate	1.1278	0.1735	0.0193
O-Toluidine	1.1199	0.1633	0.0415
Enrofloxacin	1.1165	0.1590	0.0207
1-Methyl-1,3-cyclohexadiene	1.1156	0.1579	0.0208
MG (18:2(9Z,12Z)/0:0/0:0)	1.1100	0.1505	0.0028
Simulansine	1.1069	0.1465	0.0111
alpha-Terpineol propanoate	1.0921	0.1272	0.0052
Androstenol	1.0899	0.1242	0.0221
Equol	1.0856	0.1185	0.0497
Prolyl-Tyrosine	1.0856	0.1185	0.0497
LysoPC(22:2(13Z,16Z))	1.0856	0.1185	0.0497
(4E)-1,7-bis(4-hydroxyphenyl)hept-4-en-3-one	1.0747	0.1039	0.0414
Diethylphosphate	1.0693	0.0966	0.0261
2-acetyl-1-alkyl-sn-glycero-3-phosphocholine	0.8288	-0.2710	0.0164
LPC (18:0)	0.7986	-0.3245	0.0024
Phosphorylcholine	0.7933	-0.3340	0.0443
Pedalitin	0.7243	-0.4653	0.0443
1-(5Z,8Z,11Z,14Z-eicosatetraenoyl)-sn-glycero-3-phosphate	0.7213	-0.4713	0.0260
Homoarecoline	0.7141	-0.4859	0.0471
LysoPC(P-18:1(9Z))	0.7060	-0.5023	0.0350
Uric acid	0.6726	-0.5722	0.0168
1-Arachidonoylglycerophosphoinositol	0.6652	-0.5881	0.0306
N′-Formylkynurenine	0.6542	-0.6121	0.0107
Sclareol	0.6458	-0.6308	0.0062
Graveoline	0.6339	-0.6576	0.0033

Glycerophosphocholine	0.6255	-0.6769	0.0375
LysoPC(22:5(4Z,7Z,10Z,13Z,16Z))	0.5999	-0.7372	0.0426
PC (18:5e/6:0)	0.5993	-0.7387	0.0056
PC (18:4e/4:0)	0.5862	-0.7707	0.0031
LPC (20:3)	0.5851	-0.7733	0.0427
ACar(8:1)	0.5789	-0.7886	0.0387
LysoPC(22:6(4Z,7Z,10Z,13Z,16Z,19Z))	0.5756	-0.7968	0.0060
Uric acid	0.5650	-0.8238	0.0434
LPC (22:3)	0.5551	-0.8491	0.0061
Docosapentaenoic acid (22n-3)	0.5379	-0.8945	0.0040
D-Aspartic acid	0.5177	-0.9498	0.0451
LPC (16:2)	0.4959	-1.0118	0.0331
PC (2:0/20:2)	0.4676	-1.0968	0.0027
LPC (22:4)	0.4620	-1.1139	0.0153
LPI (18:2)	0.4527	-1.1433	0.0369
Tetrahydrocortisone	0.4390	-1.1876	0.0214
Pipercyclobutanamide B	0.4283	-1.2232	0.0369
FAHFA (15:0/22:3)	0.4282	-1.2238	0.0429
LPC (22:5)	0.4248	-1.2350	0.0100
LPE (22:5)	0.4172	-1.2611	0.0196
LysoPC (20:2(11Z,14Z))	0.4164	-1.2641	0.0066
PC (14:1e/6:0)	0.3873	-1.3684	0.0119
L-Carnitine	0.3854	-1.3756	0.0005
6-Aminopenicillanic acid	0.3795	-1.3979	0.0312
LPE (20:2)	0.3716	-1.4282	0.0360
LPC (20:2)	0.3676	-1.4439	0.0004
FAHFA (18:2/22:3)	0.3480	-1.5228	0.0429
LPE (20:1)	0.3409	-1.5527	0.0176
Epinephrine	0.3166	-1.6591	0.0088
PC (22:6e/2:0)	0.3105	-1.6873	0.0016
LysoPE (20:1(11Z)/0:0)	0.2802	-1.8355	0.0136
FAHFA (22:6/22:3)	0.2782	-1.8460	0.0216

Supplementary Table S2. The differentially abundant metabolites enriched to the pathway by KEGG analysis.

KEGG pathway	Metabolites
Choline metabolism in cancer	Phosphorylcholine<br>Glycerophosphocholine<br>lysophosphatidylcholine (LPC)
Glycerophospholipid metabolism	Phosphorylcholine<br>Glycerophosphocholine<br>Lysophosphatidylcholine (LPC)

PPAR signaling pathway	Alpha-dimorphecolic acid
D-Amino acid metabolism	L-Proline<br>D-Aspartic acid
Arginine and proline metabolism	L-Proline<br>Creatinine
Adrenergic signaling in cardiomyocytes	Epinephrine
Regulation of lipolysis in adipocytes	Epinephrine
Bile secretion	L-Carnitine<br>Uric acid
Renin secretion	Epinephrine
Thermogenesis	L-Carnitine
Ether lipid metabolism	Glycerophosphocholine
cAMP signaling pathway	Epinephrine
Linoleic acid metabolism	Alpha-dimorphecolic acid
Alanine, aspartate and glutamate metabolism	D-Aspartic acid
Mineral absorption	L-Proline

Supplementary Table S3. The relative expression abundance of gut microbiota at the phylum level.

Phylum	WT1	WT2	WT3	KO1	KO2	KO3
p_Firmicutes	55.53	51.24	53.09	90.74	41.00	41.07
p_Bacteroidota	2.58	17.37	24.42	0.63	9.33	29.96
p_Verrucomicrobiota	0.89	11.05	1.95	7.39	39.06	3.72
p_Actinobacteriota	34.45	9.86	10.92	0.56	8.79	4.03
p_Desulfobacterota	0.36	4.99	1.79	0.12	0.16	12.51
p_Campylobacterota	5.60	0.58	5.14	0.03	0.18	7.12
p_Proteobacteria	0.49	4.56	1.92	0.48	1.42	0.59
p_Patescibacteria	0.06	0.04	0.03	0.03	0.03	0.39
p_Deferribacterota	0.03	0.01	0.61	0.01	0.00	0.50
p_unclassified	0.00	0.25	0.08	0.00	0.02	0.10
p_Cyanobacteria	0.00	0.05	0.00	0.01	0.00	0.00
p_Spirochaetota	0.01	0.00	0.05	0.00	0.00	0.00

Supplementary Table S4. Top 30 of the differentially abundant gut microbiota at the genus level.

Genus	WT1	WT2	WT3	KO1	KO2	KO3
Muribaculaceae_unclassified	1.90	9.50	16.03	0.45	8.73	25.71
Lactobacillus	18.15	1.16	9.33	1.28	24.90	12.78
Allobaculum	12.07	15.78	4.24	49.61	1.46	0.05
Ligilactobacillus	1.70	0.52	2.73	3.75	0.61	0.55
Akkermansia	0.56	11.05	1.95	7.39	39.06	3.72
Dubosiella	3.74	9.04	1.51	14.96	0.08	3.41
HT002	6.18	0.26	8.35	0.55	7.41	1.84
Coriobacteriaceae_UCG-002	17.39	8.05	3.79	0.20	4.00	2.11

Bifidobacterium	16.29	0.85	6.70	0.10	4.53	1.07
Muribaculum	0.11	1.55	1.91	0.03	0.24	2.07
Lachnospiraceae_unclassified	0.77	2.07	2.51	2.35	0.52	6.38
Helicobacter	5.60	0.58	5.14	0.03	0.18	7.12
Desulfovibrio	0.20	0.77	0.31	0.12	0.13	12.38
Lachnospiraceae_NK4A136_group	0.54	0.64	1.01	0.48	0.52	4.02
Eubacterium_coprostanoligenes_group_unclassified	0.11	1.60	5.14	3.67	0.37	0.02
Clostridia_UCG-014_unclassified	0.19	2.05	0.55	0.03	0.41	1.21
Parasutterella	0.31	4.27	1.75	0.25	0.69	0.54
Bacteroides	0.22	5.29	2.12	0.02	0.08	0.09
Dorea	1.80	0.51	3.62	0.88	1.17	0.06
Sellimonas	0.42	0.81	0.87	4.04	0.09	0.00
Alistipes	0.13	0.14	0.18	0.01	0.10	0.05
Faecalibaculum	3.83	1.85	0.31	0.01	0.43	0.01
Lactococcus	0.39	1.17	0.33	2.96	0.30	0.00
Desulfovibrionaceae_unclassified	0.16	4.24	1.56	0.01	0.03	0.11
Clostridiales_unclassified	0.26	0.33	1.29	0.07	0.17	1.94
Parabacteroides	0.06	0.32	2.42	0.01	0.02	0.56
Paramuribaculum	0.01	0.00	0.01	0.01	0.00	0.59
Enterorhabdus	0.13	0.12	0.13	0.20	0.17	0.24
Firmicutes_unclassified	0.20	2.18	0.79	0.11	0.33	1.46
Candidatus_Saccharimonas	0.06	0.04	0.03	0.03	0.03	0.39
Others	6.54	13.25	13.42	6.39	3.25	9.52

Supplementary Table S5. Primer sequences for real-time qRT-PCR detection.

Genes	Primer	Sequence 5′–3′
Mouse CHI3L1	Forward	ATGCACACCTCTACTGAAGCC
	Reverse	ACCAGCTTGTACGCAGAGC
Mouse Col1a1	Forward	CTGGCGGTTCAGGTCCAAT
	Reverse	TTCCAGGCAATCCACGAGC
Mouse GDF15	Forward	CAGTCCAGAGGTGAGATTGGG
	Reverse	CAGGCGTGCTTTGATCTGC
Mouse AKR1B10	Forward	TGACACCTTCTCGAATACAGGA
	Reverse	ATTCCGCATGGAAAGGGAAGT
Mouse ACTA2	Forward	GGCACCACTGAACCCTAAGG
	Reverse	ACAATACCAGTTGTACGTCCAGA
Mouse IL-6	Forward	TCTATACCACTTCACAAGTCGGA
	Reverse	GAATTGCCATTGCACAACTCTTT
Mouse IL-1α	Forward	TGATGAAGCTCGTCAGGCAG
	Reverse	AGGTGCACCCGACTTTGTTC
Mouse Il-1β	Forward	GCAACTGTTCCTGAACTCAACT
	Reverse	ATCTTTTGGGGTCCGTCAACT
Mouse β-actin	Forward	GGCTGTATTCCCCTCCATCG

	Reverse	CCAGTTGGTAACAATGCCATGT
Mouse SIRT2	Forward	CCACGGCACCTTCTACACATC
	Reverse	CACCTGGGAGTTGCTTCTGAG
Human SIRT2	Forward	CACGCAGAACATAGATACCCTG
	Reverse	CAGTGTGATGTGTAGAAGGTGC
Human β-actin	Forward	CATGTACGTTGCTATCCAGGC
	Reverse	CTCCTTAATGTCACGCACGAT

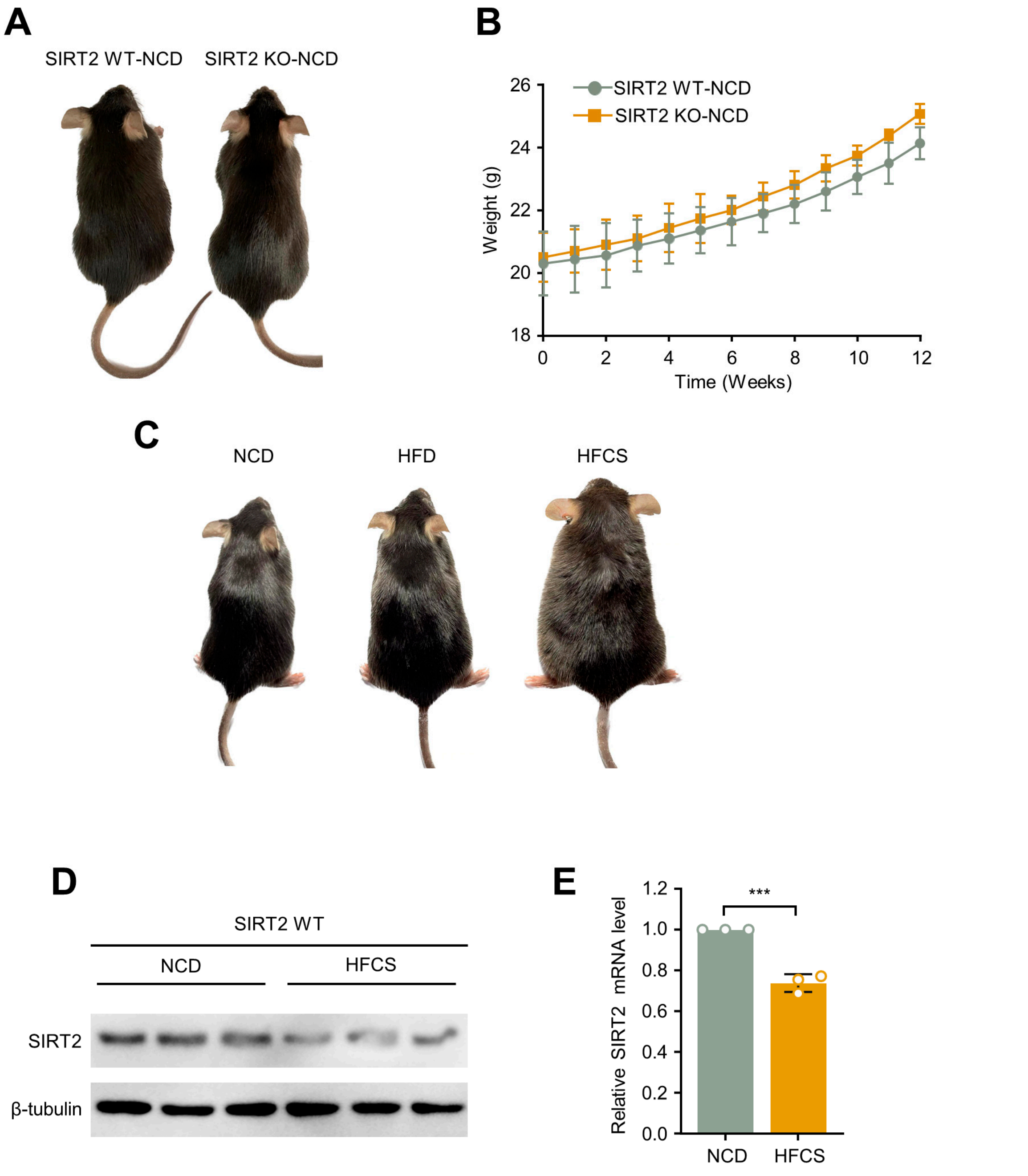

Figure S1. HFCS diet induced mice obesity. (A) The representative gross morphology and (B) the body weight of SIRT2 WT and SIRT2 KO mice with normal chow diet (NCD) for 12 weeks. (C) The representative gross morphology of SIRT2 WT mice fed with NCD, high-fat diet (HFD), or high-fat/high-cholesterol/high-sucrose (HFCS) diet for 12 weeks (D) Western blotting of the liver tissues from the SIRT2 WT mice with NCD or HFCS diet for 12 weeks to detect SIRT2, and three

representative mice from each group were selected for this experiment. (E) The mRNA levels of SIRT2 were analyzed in liver from SIRT2 WT mice with NCD or HFCS diet for 12 weeks (N=9/per group). ***$p < 0.001$.

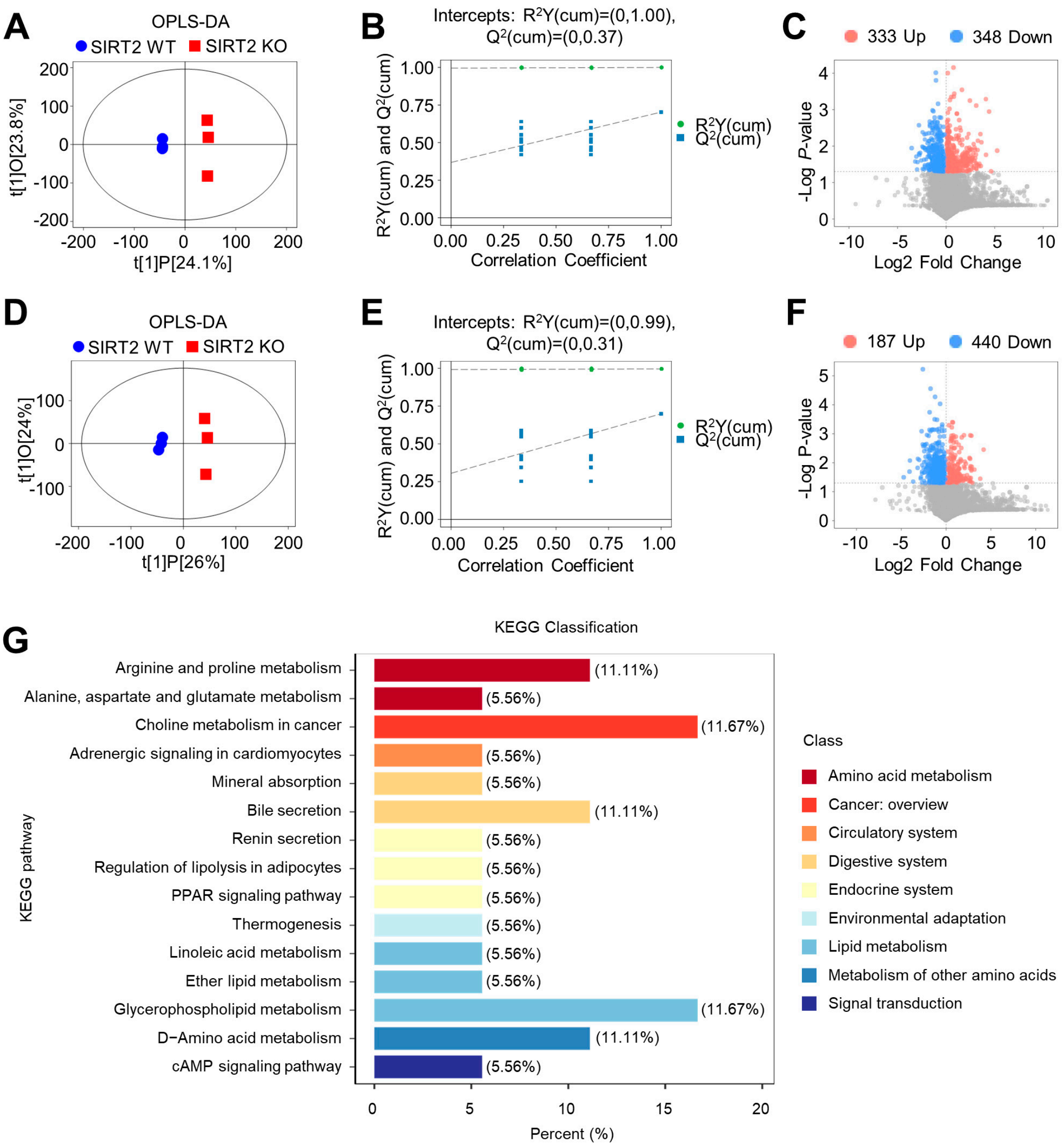

Figure S2. SIRT2 deficiency modulated serum metabolome in mice with HFCS diet for 12 weeks. Orthogonal projections to latent structures discriminant analysis (OPLS-DA) score plot was derived from metabolomics data tested in (A) positive and (D) negative ion mode, respectively. The OPLS-DA models of the (B) positive and (E) negative ion mode were validated by permutation test. Volcano plot shows the metabolites that were different between SIRT2 WT and SIRT2 KO mice, detected by the (C) positive ion and (F) negative ion mode. (G) KEGG analysis was conducted using all of the differential metabolites from both positive and negative ion modes (N=3/per group).

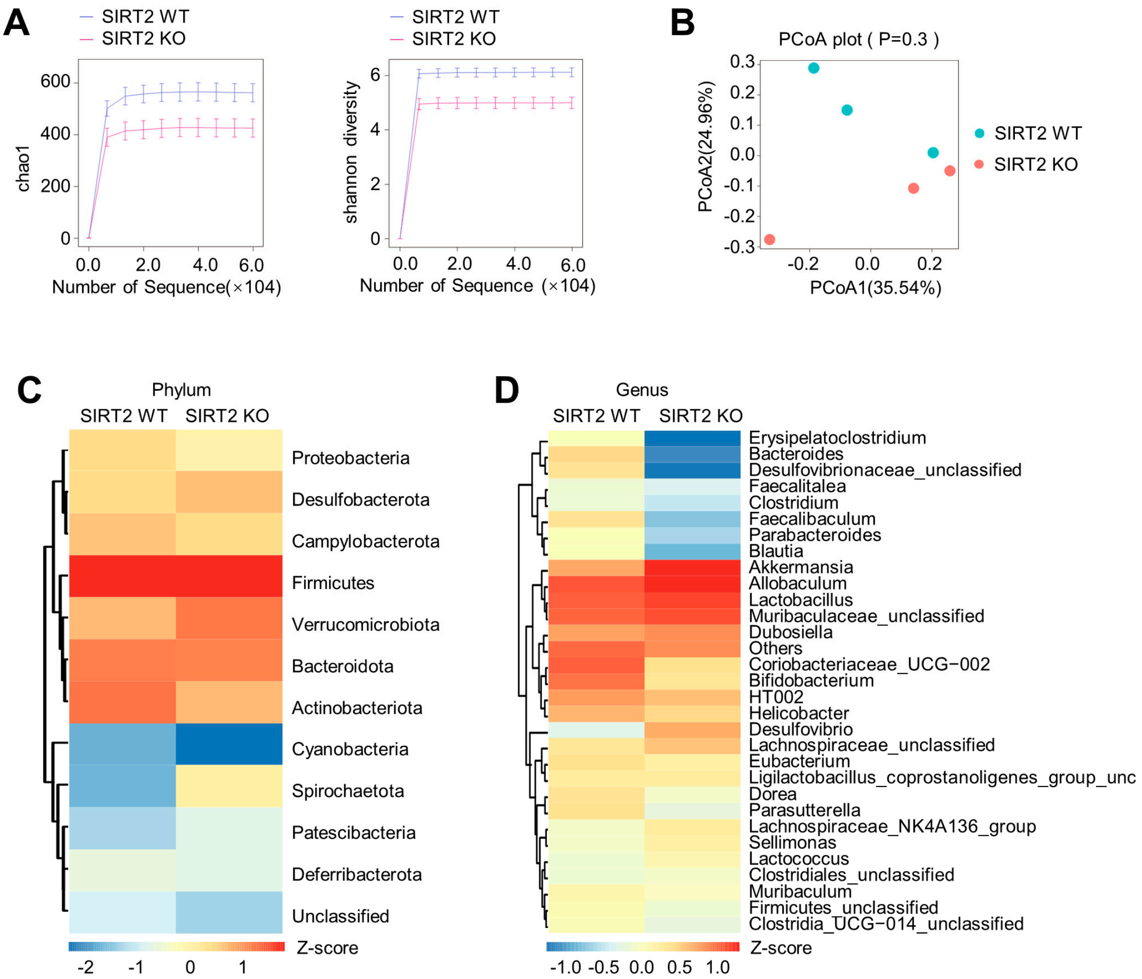

Figure S3. Diversity and composition analysis of gut microbiota in mice with HFCS diet for 12 weeks. (A) Chao1 and Shannon indexes respectively indicated the richness and diversity of the gut microbiota (N=3/per group). (B) Principal component ordination analysis (PCoA) chart of gut microbiota in mice with HFCS feeding for 12 weeks (N=3/per group). (C) Heatmap of hierarchical clustering analysis of gut microbiota at the phylum level in SIRT2 KO and SIRT2 WT mice (N=3/per group). (D) Heatmap of hierarchical clustering analysis of the top 30 gut microbiota at the genus level in SIRT2 KO and SIRT2 WT mice (N=3/per group). The "_unc" means "_unclassified".

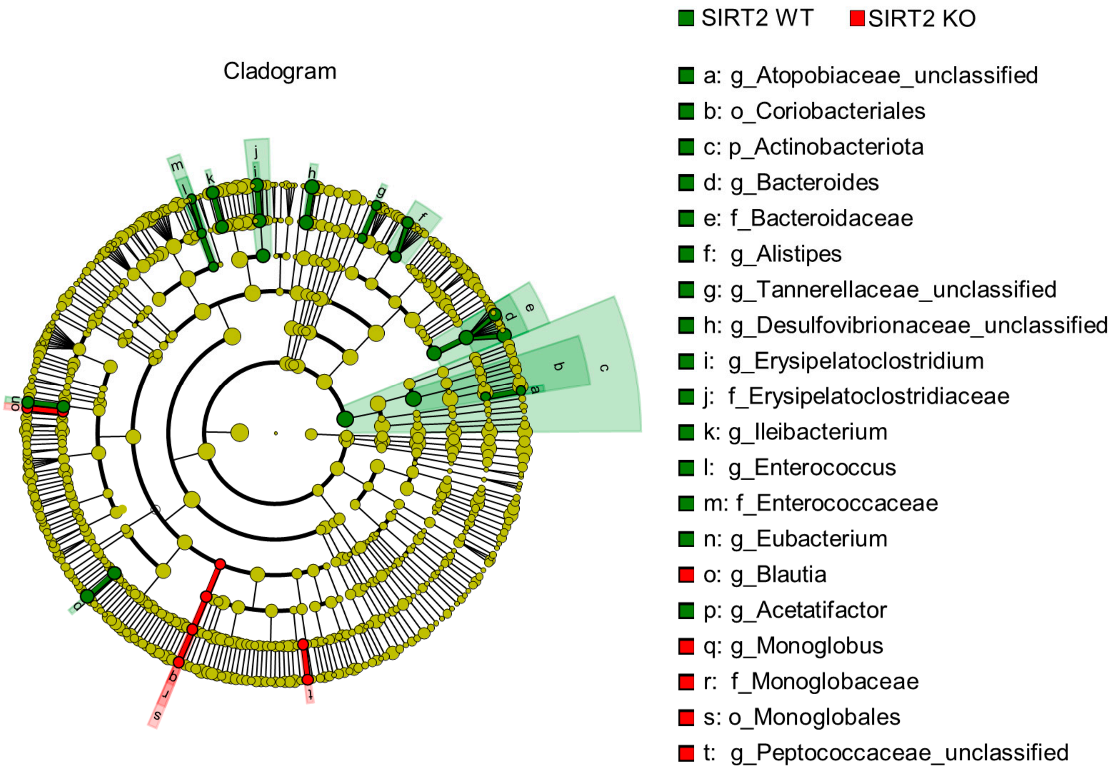

Figure S4. SIRT2 deficiency promoted the intestinal microbiota dysbiosis. Taxonomic cladogram generated by LEfSe analysis showed the taxa significantly enriched in the SIRT2 WT (green), and SIRT2 KO (red) mice. The circles from inside to outside represent taxonomic levels from domain to species, and the size of each dot represents the relative abundance of the corresponding taxon (N=3/per group).

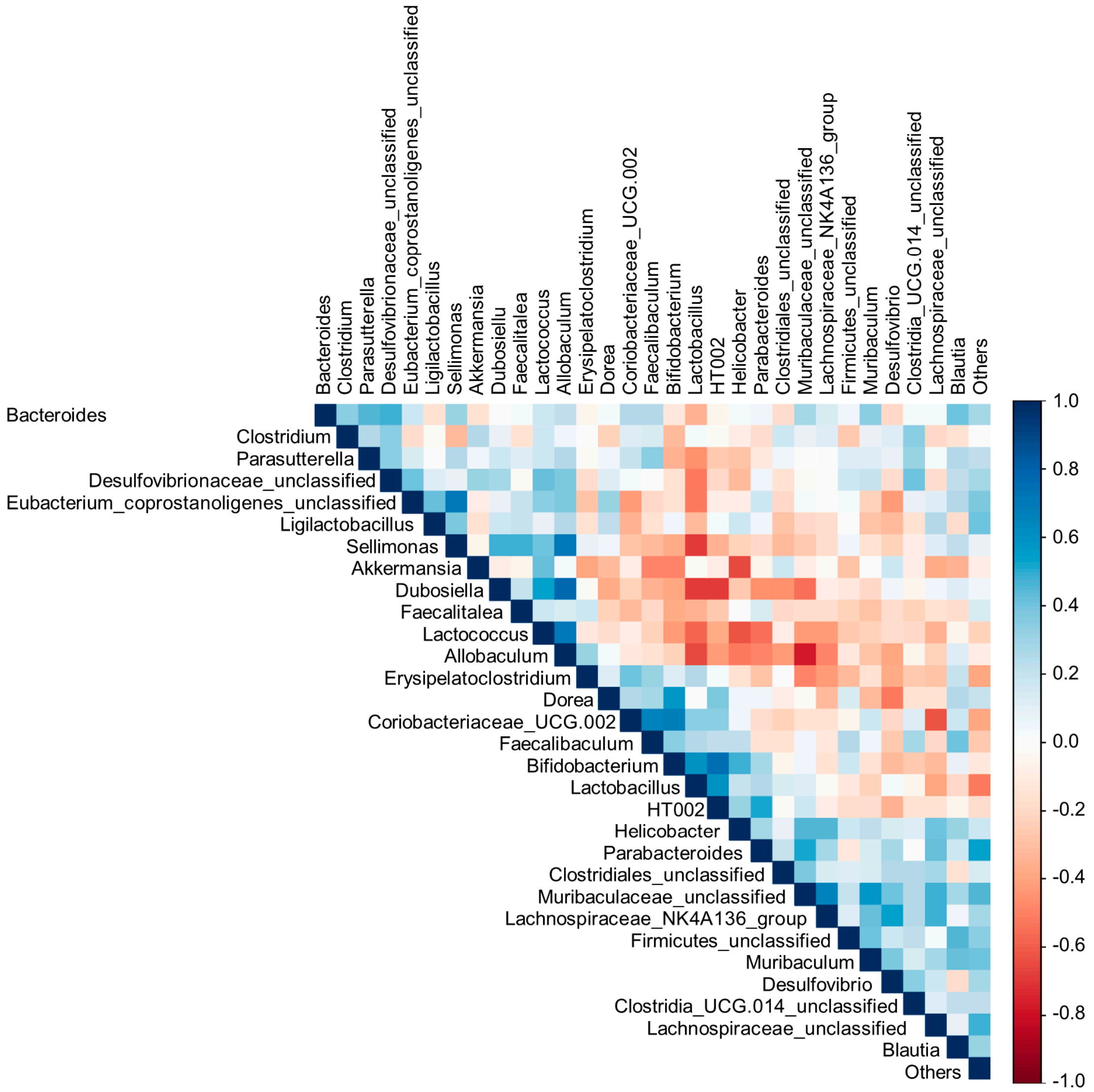

Figure S5. Correlation analysis of differential gut microbiota between SIRT2 KO and SIRT2 WT NAFLD mice. Spearman correlation analysis among gut microbiota at genus levels between the SIRT2 KO and SIRT2 WT groups. The correlation analysis value is represented by the colors of grinds. Blue represents positive correlation, and red represents negative correlation (N=3/per group).
